# The Influence of Safety Performance on Competitive Advantage in the Shipping Industry: A Systematic Literature Review Study

**DOI:** 10.12688/f1000research.163658.3

**Published:** 2025-08-28

**Authors:** Bharto Ari Raharjo, Hamidah Nayati Utami, Tri Wulida Afrianty, Ika Ruhana

**Affiliations:** 1 Brawijaya University, Malang, East Java, Indonesia

**Keywords:** Safety Performance, Systematic Literature Review, Occupational Safety, Competitive Advantage, Shipping Industry

## Abstract

This study analyzes research trends and developments related to *Safety Performance* and its implementation solutions through *the Systematic Literature Review* (SLR) based on Scopus data from 1919 to 2023. The results showed a significant increase in publications, especially after 2000, reflecting global attention to safety issues. The main factors that affect *Safety Performance* include the safety culture, technology, human factors, and regulations. Key challenges include resource constraints, inconsistent regulations, and reliance on legacy technologies. Strategic solutions include strengthening safety culture, adopting modern technology, and simulation-based training. This study also highlights the role of *Safety Performance* in creating *a competitive advantage* through improved reputation, efficiency, and sustainability. These findings are relevant for supporting occupational safety, particularly in the Indonesian shipping industry.

## 1. Introduction

In the world of shipping, safety is not only a moral responsibility but also a source of competitive advantage. Shipping companies that are able to demonstrate a high commitment to safety have a greater chance of winning customer trust, expanding market share, and reducing operational costs owing to avoidable incidents. Based on a report by Allianz Global Corporate & Specialty, ^1^ 75% of financial losses arise from the top 10 causes of loss, while the top three causes account for close to half (45%) of the value. By prioritizing safety, companies can create a positive reputation and ensure business sustainability amid fierce industry competition.

In the context of shipping safety, the Ministry of Transportation, through the Directorate General of Sea Transportation, continues to campaign for the importance of shipping safety to the public. Shipping safety includes the fulfillment of safety and security requirements in water transportation and ports. The shipping community and sea transportation actors are expected to become increasingly aware of the importance of safety planning from the beginning to reduce the risk of accidents.

Based on data from the Directorate General of Sea Transportation, there were a total of 1,453 ship accident incidents in the 2015–2023 period. Of these, 37% were sunken ships, 28% were shipwrecked, and the remainder included fires, collisions, and other incidents. The trend of shipsinking accidents shows an average annual decline of 8%. However, incidents such as ship fires showed fluctuations, with a peak of 56 cases occurring in 2018. These data demonstrate the importance of collaborative efforts between regulators, cruise operators, and the public to improve safety.

Previous studies have provided a solid foundation for improving the shipping safety. Grech, Horberry, and Koester ^2^ in the book Human Factors in the Maritime Domain highlight the importance of human factors in improving shipping safety, especially in decision-making and operational risk management. Safety Culture Indicators to show that a strong safety culture can significantly reduce shipping incidents. ^3^ Meanwhile, Ekstrand and Tapaninen ^4^ evaluated the role of technologies such as the Automatic Identification System (AIS) and found that the implementation of this technology can reduce the risk of incidents by up to 30%. Hale et al. ^5^ proposed a systemic approach to safety management, emphasizing the need to integrate training, regulations, and periodic inspections to create a holistic approach that is more effective than fragmented methods. Huang et al. ^6^ demonstrated the potential of big data technology and predictive analytics to predict operational risks and provide early warnings to reduce the likelihood of incidents. These studies emphasize the importance of multidimensional collaboration in improving shipping safety, from strengthening safety culture to applying advanced technology. The complexity of this problem demonstrates the need for a systemic and holistic approach to understanding and improving shipping safety.

To address these challenges, this study used a Systematic Literature Review (SLR) approach. SLR is designed to identify, assess, and interpret research findings related to a particular topic. ^7^ This approach allows researchers to analyze the implementation of Safety Performance in depth from a theoretical and practical perspective, as well as identify relevant solutions to address shipping safety problems in Indonesia. Thus, the SLR is an important and urgent step in developing an evidence-based framework for improving marine transportation safety.

## 2. Literature review

### 2.1 Systematic Literature Review (SLR)

*Systematic Literature Review* is a method that is currently widely used by researchers and academics to review scientific literature. This is because the SLR method can avoid bias and a subjective understanding of the research. ^7^ SLR has been proven to be a method that can effectively provide an overview of research trends, methodologies, and *research coverage fields* in previous studies. ^8, 9^

A Systematic Review (SR), often referred to as a Systematic Literature Review (SLR), is a structured method used to collect, critically evaluate, integrate, and present findings from various research studies focused on a specific research question or topic. ^10^ This approach offers a broader and more accurate understanding than traditional literature reviews. ^11^ The main objectives of an SLR include identifying, examining, evaluating, and interpreting all available studies related to the topic or phenomenon of interest, as well as the relevant research questions. ^12^ Moreover, an SLR enables researchers to gain deep insight and analytical capability regarding the research topic while providing a comprehensive overview of the background for developing scientific work. ^13^

### 2.2 Safety performance

*Safety Performance* is the performance of workers who prioritize safety at work by following applicable safety and health rules at work. ^14^ Based on the understanding provided by experts, the operational definition of *Safety Performance* is obtained, namely, the performance of a company in prioritizing work safety by following applicable rules. Brand ^15^ states that *the performance* component shows large behavioral dimensions that are relevant to a given task. This model combines two dimensions of *Safety Performance*: compliance and participation. Compliance involves and adheres to safety procedures and carries out work in a safe way, preparing and using the right safety equipment at work. Meanwhile, participation is an individual involvement that is not directly related to individual safety, but can also support the realization of a safe environment.

Safety performance can be defined as the level of safety related to work within an organization that enhances resilience and reduces the risk of accidents. ^16^ Safety performance is also closely related to psychological factors, such as employees’ motivation to behave safely and their perception of a safe work environment. ^17^ Furthermore, safety performance involves activities or behaviors exhibited by individuals at the workplace to improve health and safety for employees, customers, the community, and the environment. ^18^ Safety performance can be measured based on three main aspects: safety inspection, accident investigation, and safety training. ^19^

## 3. Research methods

This study uses the *Systematic Literature Review* (SLR) method with data collected through a literature search of the Scopus database. As shown in Table 1, the data on safety performance publications was sourced from the Scopus database and processed by the researcher (2025). 

The search results in the SCOPUS database were carried out through two main steps: an overall search across the SCOPUS database and a more specific search on the *subject area* Business, Management, and Accounting. In the first step, a search was conducted using the keywords (“safety*” AND “performance*”), which resulted in 190,532 articles from 1919 to 2023. All the articles were written in English and covered a wide range of research areas. The second step was to narrow the search to the *subject area* Business, Management, and Accounting using the keywords (“safety*” AND “performance*”) AND (LIMIT-TO (SUBJAREA, “SPARK PLUG”). This search yielded 4,769 articles written in English, with a publication time span from 1963 to 2023. This step provides a sharp focus on the relevance of the article to the field being studied.

The eligibility criteria in this study include data collection related to factors that affect safety performance, such as safety culture, technology, human factors, and regulations. The exclusion criteria for this study include manuscripts that are not fully accessible and those that do not align with the research topic. The risk assessment of bias is conducted using standardized tools to evaluate potential bias in study design, data collection methods, and potential conflicts of interest, with independent assessors working together to reach consensus. Synthesis of results is done by combining data from relevant studies using meta-analysis if possible, or synthesizing narratively. Reporting bias assessments are performed to ensure that the reported results reflect accurate data, with no results being ignored or only reported if significant. Finally, certainty assessments were conducted using the GRADE approach to assess the quality of evidence involving risk of bias, consistency of findings, accuracy of estimates, and potential conflicts of interest.

The journals found in the search used both qualitative and quantitative approaches, which showed that research related to *Safety Performance* has remained relevant in recent years. This relevance is reflected in the diversity of the research methods used to explore various aspects of *Safety Performance.* As shown in Table 2, the steps and objectives of the research using *Systematic Literature Review analysis*:

The literature review *questions* formed are as follows.

1) What is the map for the development of research on *Safety Performance* in various parts of the world?
2) How is the Development of Discourse in research on *Safety Performance*?
3) What are the key factors that affect Safety Performance in the shipping industry?
4) What are the main challenges faced by shipping companies in implementing Safety Performance?
5) What solutions can be adopted to improve Safety Performance in Indonesian shipping companies?
6) What is the role of Safety Performance in creating Competitive Advantage for shipping companies?

## 4. Results

Before discussing the diagram, it is essential to outline the study selection process, which involves identifying records, screening for eligibility, and selecting studies based on inclusion criteria. The diagram below shows the step-by-step process, from initial identification to the final selection of studies included in the review.

**Figure 1.**
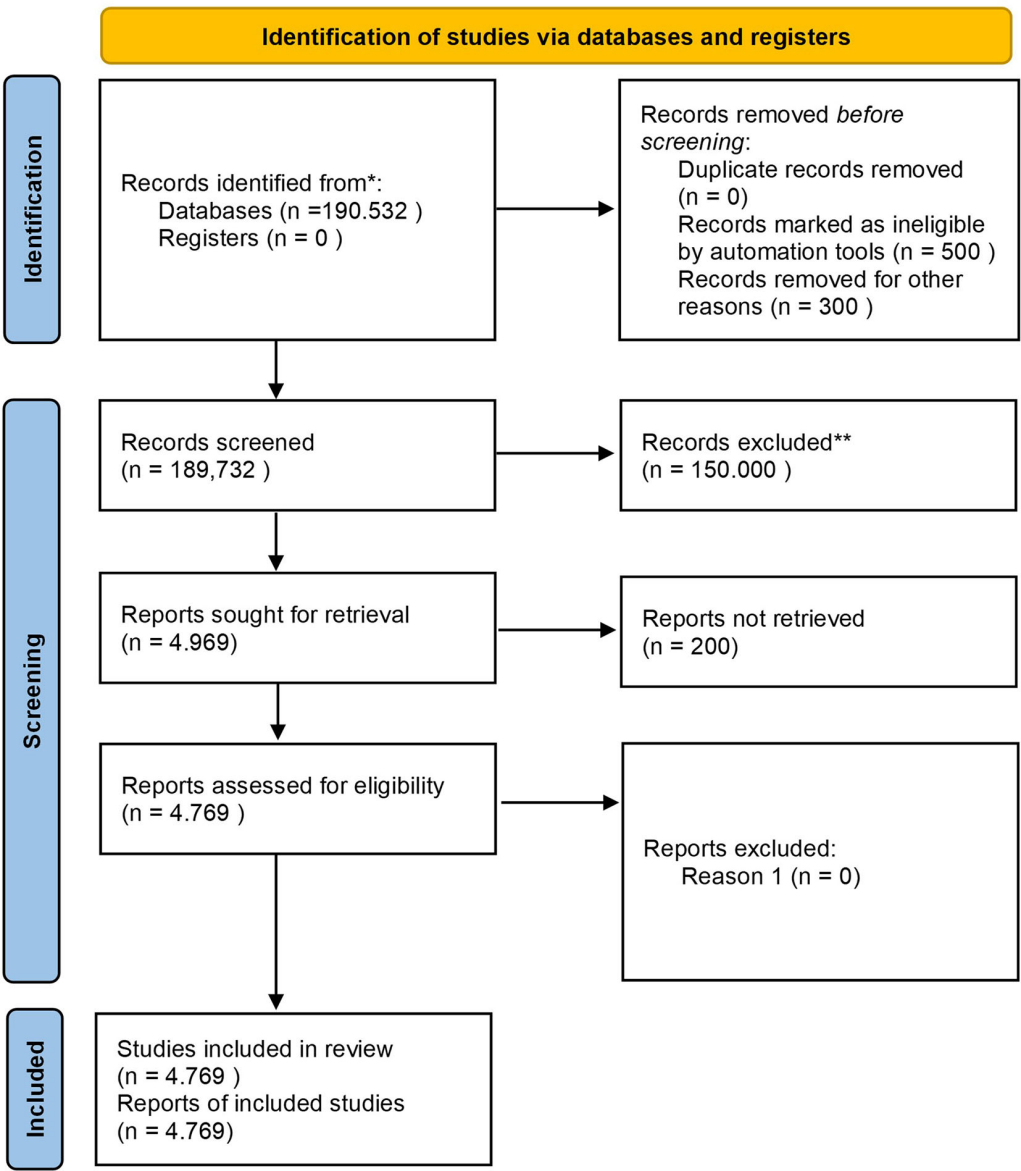
PRISMA 2020 flow diagram systematic literature reviews.

Based on Figure 1, the study selection process began with a comprehensive search through the Scopus database, which resulted in a total of 190,532 documents. After removing duplicates, ineligible records, and irrelevant studies, 4,969 reports were requested to be retrieved, but 200 reports could not be retrieved due to access or availability issues, resulting in only 4,769 reports being assessed for eligibility, resulting in 4,769 studies being included in the final review. The included studies focus on different aspects of Safety Performance in the context of Business, Management, and Accounting, with study characteristics that include quantitative and qualitative types, as well as coming from various industry sectors such as manufacturing, transportation, and healthcare. The study covers a range of publications from 1963 to 2023.

**Table 1.** Safety performance publication data.

Description	All Documents *Safety Performance*	Article *Business, Management and Accounting*
Search Engines	www.scopus.com	www.scopus.com
Limitation	None	BUSI
Year	1919-2023	1963-2023
Number of Documents	190,532 documents	4,769 documents

Source: Processed by Researcher (2025).

A risk of bias assessment was conducted on each study, which included an evaluation of selection bias, performance, detection, and reporting, with results showing that some studies had potential bias in reporting unbalanced outcomes. Each study provides summary statistics and effect measures that calculate the influence of factors such as safety culture, technology, and human factors on safety performance. The results of the synthesis show that safety culture has a significant influence on safety performance, while the influence of technology is more varied.

Heterogeneity investigations showed that study design, industry type, and cultural factors influenced the results, while sensitivity analysis showed that results for safety culture remained consistent even though studies with a high risk of bias were excluded. An assessment of reporting bias revealed that some studies tended to report positive results that could lead to overestimation of effects. Certainty assessment of the evidence with the GRADE approach showed that the evidence for safety culture and technology adoption had a moderate level of certainty, while the evidence for human factors had low certainty due to methodological inconsistencies and a higher risk of bias.

## 5. Discussion

**Figure 2.**
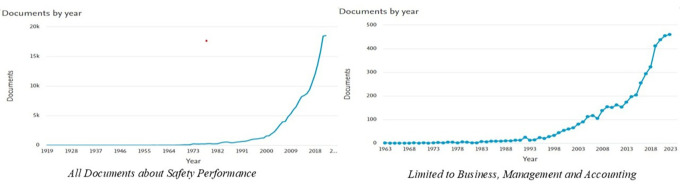
Number of documents per year related to *safety performance.*

The results of this study show that the number of documents related to *Safety Performance* has experienced a significant increasing trend over time. Based on the data processed from Scopus, the graph of document distribution per year ( Figure 2) shows two main patterns.

**Table 2.** Steps and objectives *of systematic literature review.*

No	Step	Purpose
1	Search and retrieve data from publication search engines	Obtain publication data related to research topics
2	Analyze reference data	Understanding the background and context of the research landscape
3	Analyze the title and abstract	Quickly understand the scope of research
4	Conduct an in-depth reading of selected articles	Understand the discourse, theories/concepts, methodologies used and their findings
5	Conducting mapping and analysis of discourses, theories/concepts, research methodologies and findings	Understand the trends in research developments for related topics
6	Mapping research gaps	Identifying opportunities for further study

Source: Processed by Researcher (2025).

Figure 2 shows the distribution of the number of documents related to *Safety Performance* per year based on data from Scopus. The graph on the left depicts the overall number of documents, whereas the graph on the right focuses on documents in the *Business, Management and Accounting categories.* Based on the graph on the left, the number of publications on *Safety Performance* shows a significant increase, particularly after 2000. Previously, publications included only a few documents per year, from 1919 to the 1990s. However, this trend changed, with a sharp spike in the early 2000s, reaching nearly 20,000 documents per year by 2023. This reflects the increasing attention paid to safety issues in various sectors, along with technological developments and the need for safer working practices.

Meanwhile, the graph on the right shows that research related to *Safety Performance* in the fields of *Business, Management and Accounting* is also experiencing a similar upward trend, albeit on a smaller scale. Publications became consistent after the 1980s and experienced a sharp spike after the 2000s, reaching more than 400 documents per year by 2023. Overall, these two graphs show that *Safety Performance* is becoming an increasingly relevant topic in various disciplines and industrial contexts.

**Figure 3.**
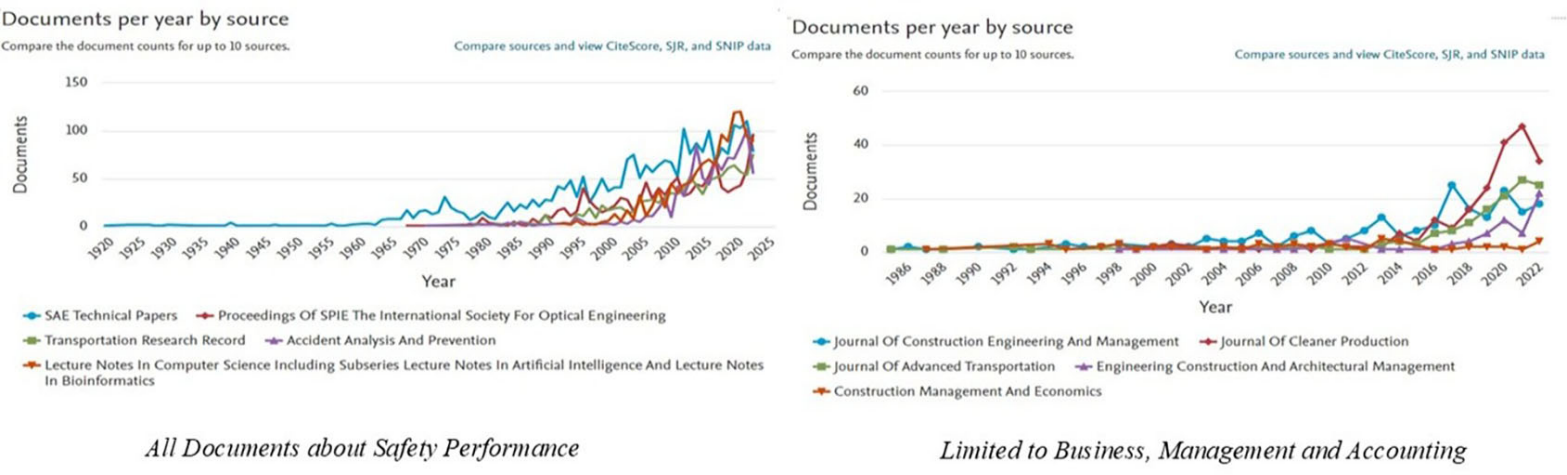
Top 10 safety performance publication sources.

Figure 3 shows the ranking of the top ten publication sources that contributed to *Safety Performance research*, both in general and in the specific categories *of Business, Management and Accounting.* In the graph on the left, it can be seen that general publications on *Safety Performance* are dominated by engineering and transportation journals, such as *SAE Technical Papers*, *Proceedings of SPIE*, and *Transportation Research Record.* This trend showed a significant increase after 2000, reflecting the dominance of engineering and science in safety-related research. The number of documents from these journals has increased sharply over the past decade, indicating increased attention to safety issues in various technical sectors.

Meanwhile, the graph on the right highlights the sources of publications that focus on *the Business, Management and Accounting* categories. Journals such as *the Journal of Construction Engineering and Management*, *the Journal of Cleaner Production*, and *Engineering Construction and Architectural Management* are the main sources of publications in this field. The trend of publications in this category has been consistent since 2000 and continues to increase, peaking in recent years. Research in this area has generally focused on project management, sustainability, resource efficiency, and technological innovation in the context of safety.

**Figure 4.**
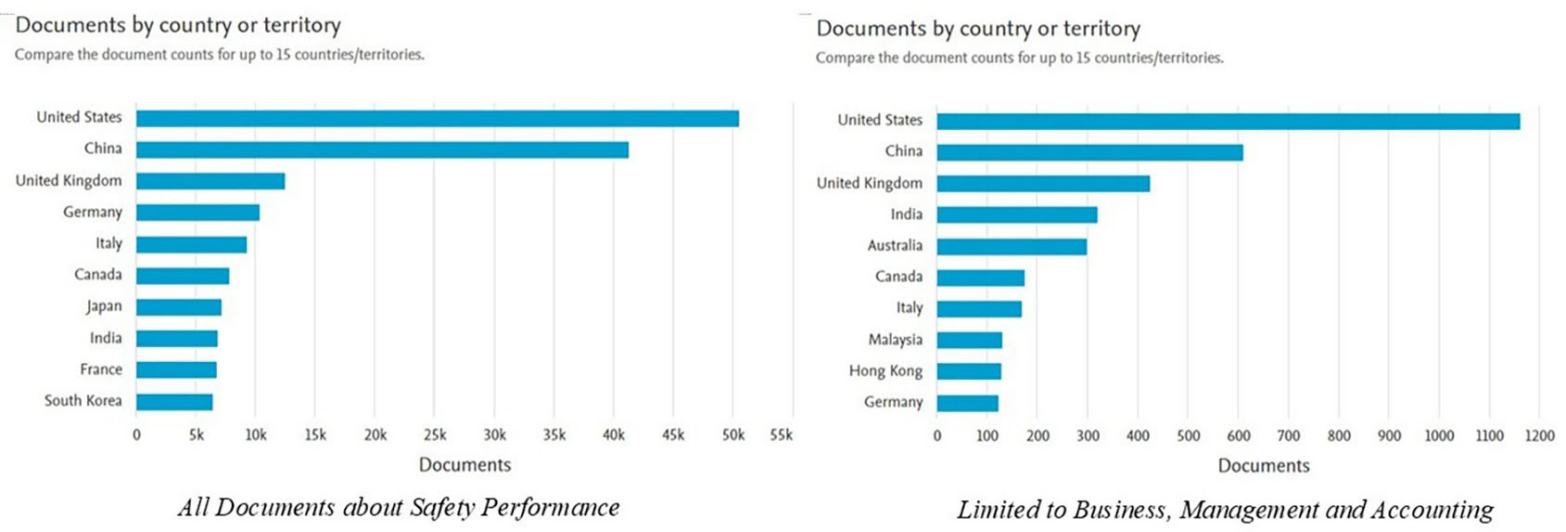
Documents by country/region.

Figure 4 shows the distribution of documents related to *Safety Performance* by country or region for both document categories and documents limited to the fields of *Business, Management and Accounting.* Here is the explanation:

In the graph on the left, which includes all documents on *Safety Performance*, it can be seen that the United States has the highest number of publications, exceeding 50,000 documents. This position is followed by China, with the number of documents approaching 40,000, as well as the United Kingdom, Germany, and Italy, each producing approximately 10,000 to 15,000 documents. Other countries such as Canada, Japan, and India also contributed significantly to the number of publications. This shows that the issue of *Safety Performance* has become a global concern, with the dominance of developed countries with strong research and development infrastructure.

The graph on the right shows the distribution of documents in the *Business, Management and Accounting* categories. The United States remains in the lead with the highest number of publications (more than 1,000 documents), followed by China, which is close to that number. Countries such as the UK, India, and Australia also make a large contribution, with a smaller but significant number of documents than other countries. Asian countries such as Malaysia and Hong Kong showed a strong presence in this category, reflecting the Asia-Pacific region's focus on safety in the context of business management and sustainability.

**Figure 5.**
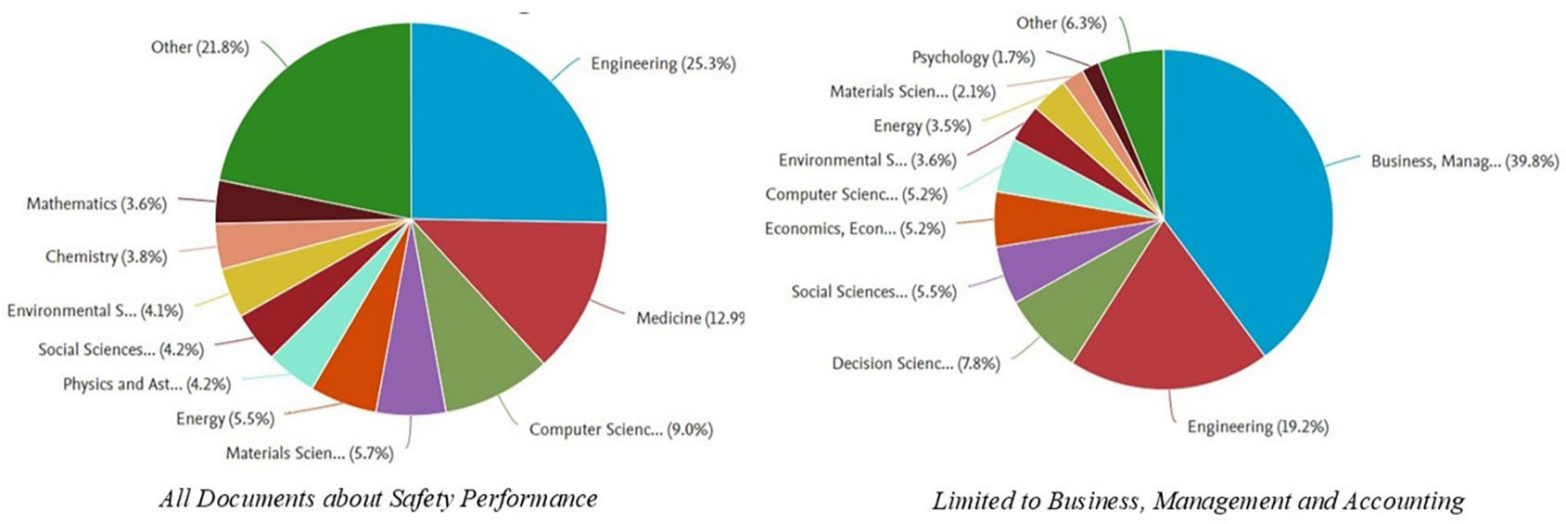
Documents by *subject area.*

Figure 5 shows the distribution of documents related to *Safety Performance* by *subject area*, both as a whole and limited to Business *, Management and Accounting category.* Here is the explanation:

In the graph on the left, which includes all documents related to *Safety Performance*, the field of engineering dominates, with a contribution of 25.3% of the total documents. Other fields that also made a significant contribution were medicine (15.0%), Computer Science (9.0%), and energy (5.5%). In addition, fields such as Social Sciences (4.9%) and environmental sciences (4.2%) also played an important role in this research. The dominance of the engineering field indicates that safety issues are generally rooted in technology and technical implementation in various industrial sectors.

Meanwhile, in the graph on the right, which focuses on the *Business, Management and Accounting* category, the Business, Management and Accounting fields are the most dominant, with a contribution of 39.8%. Engineering contributed 19.2%, reflecting the relationship between safety management and technical applications. Other fields, such as Decision Science (7.8%) and economics (5.2%), showed that research in this category also involves analytical and economic aspects. Fields such as Environmental Science and Psychology have made smaller contributions but remain relevant in understanding safety from a sustainability and behavioral perspective.

As a result of an analysis of the available literature, answers to the *literature review questions* were formulated based on the findings of previous research and the latest trends in *Safety Performance.* These answers provide in-depth insights into the key factors that affect shipping safety, challenges faced, and relevant solutions to implement. The results of the answers to *the literature review* questions are as follows.

### 1) Safety Performance Movement Map in various parts of the world

The results of *the Systematic Literature Review* show that *Safety Performance* has become a broad and relevant research topic in various parts of the world. This study covers a wide range of geographical, cultural, and industrial contexts. Detailed information on the distribution of research in different regions is presented in Table 3, which provides an overview of the global contribution to *safety* performance-related studies. The table also helps to identify areas of primary focus of the study, as well as potential areas that require further attention.

**Table 3.** *Safety performance* movement in various parts of the world.

Country	Findings
*United States*	Muhieddine *et al.* ^20^ conducted research on the development of processes to improve *Safety Performance* in the health sector. Ashraf & Pyrialakou ^21^ Traffic Safety Planning to Improve *Safety Performance* in the Public Environment *.*
China	Zhang *et al.* ^22^ conducted research on *Safety Performance* on the performance of sea piers *.* Zhong *et al.* ^23^ conducted research to develop tools that have high safety and performance.
*United Kingdom*	Erten *et al.* ^24^ developing employee occupational health and safety training methods. Rojas & Khan ^25^ conducted research to improve battery performance and safety.
Jerman	Schurmann *et al.* ^26^ developed a new approach to improve *Safety Performance controllers.* Ricker *et al.* ^27^ developing a probability-based safety method based on the reliability problem feature.
Italia	Russel *et al.* ^28^ conducted research on the efficacy and safety of eye disease healing techniques. Gore *et al.* ^29^ establishes a standard of care that is safe for patients.

Source: Processed by Researcher (2025).

Table 3 shows that *Safety Performance* research in developed countries covers various sectors according to local needs. In *the United States*, research focuses on health and transportation, with attention to the development of occupational safety processes ^20^ and traffic safety planning. ^20^ In *China*, research highlights operational efficiencies, such as *Safety Performance* at sea docks ^22^ and the development of high-security tools. ^23^

*The United Kingdom* focuses on occupational safety training ^24^ and improving battery performance. ^25^ In *Germany*, Technology-based approaches dominate Germany, such as new safety controllers ^26^ and probability-based safety methods. ^27^ Meanwhile, research in *Italy* highlights safety in health, such as eye healing techniques ^28^ and the standard of care for patients. ^29^

### 2) Development of Discourse in Research on *Safety Performance*

The results of the *Systematic Literature Review* show that *Safety Performance* has been researched through various discourses and theoretical developments, as presented in Table 4. These studies include diverse theoretical approaches such as organizational theory, behavioral theory, and risk management theory. This discourse reflects the evolution of the academic understanding of *Safety Performance*, starting from the individual aspects of the overall organizational system. Table 4 presents a summary of the theoretical developments used in the research, providing an overview of the conceptual foundations that support studies related *to Safety Performance* as well as the underlying theoretical development trends and directions.

**Table 4.** Discourse and development of *safety performance research.*

Discourse	Development
*Safety Culture*	• Niskanen ^30^: attitudes towards safety in organizations, changes in work demands, appreciation/appreciation. • Vinodkumar & Bhasi ^31^: management, knowledge, employee compliance, commitment to the south, environmental security, emergency preparedness in the organization, and safety priorities.
*Safety Performance*	• Neal *et al.* ^32^: pengetahuan, motivasi, *safety climate*. • Xia *et al.* ^33^: unsafe worker actions, surveillance, the environment, and organizational influence.

Source: Processed by Researcher (2025).

Table 4 provides an overview of the development of discourse and research related to *Safety Culture* and *Safety Performance.* In the context of *Safety Climate*, Niskanen ^30^ highlights the importance of attitudes towards safety in organizations, which includes changes in work demands and employees’ appreciation. This study underscores the role of organizational culture in building a safe work environment. Vinodkumar and Bhasi ^31^ expanded this discourse by including elements such as management, knowledge, employee compliance, safety commitment, environmental security, emergency preparedness, and safety priorities. This shows that *Safety Climate* is not only perceptual, but also includes practical and policy aspects in the organization.

On *Safety Performance*, Neal *et al.* ^32^ defined safety performance through three main elements: knowledge, motivation, and *safety climate.* This approach highlights the importance of individual competencies and an organization's safety culture to support safety performance. Xia *et al.* ^33^ expanded this discourse by highlighting factors such as unsafe worker actions, supervision, the work environment, and organizational influence. This perspective provides a deeper understanding of how internal and external factors affect the safety performance.

### 3) Key factors affecting Safety Performance in the shipping industry

The main factors that affect the Safety Performance in the shipping industry can be categorized into several important aspects. First, the Safety Culture plays a significant role in improving safety. Lu and Tsai ^3^ pointed out that a strong safety culture can reduce the risk of ship accidents, while Reason ^34^ highlighted the concept of Just Culture, which encourages openness in reporting incidents without fear of punishment. Ek et al. ^35^ also emphasized the role of leadership in building a strong safety culture.

Second, technology and navigation systems are the key elements. Ekstrand and Tapaninen ^4^ found that the use of an Automatic Identification System (AIS) can reduce the risk of incidents by up to 30%, while Perera and Mo ^36^ emphasized the role of big data-based predictive technology in detecting potential incidents. Rothblum et al. ^37^ noted that automated navigation systems can reduce the risk of human error.

Third, human factors also play an important role. Grech, Horberry, & Koester ^2^ cite crew fatigue, lack of training, and miscommunication as the main causes of accidents. Hetherington, Flin, and Mearns ^38^ highlighted fatigue as a major risk factor, whereas Schager ^39^ emphasized the importance of simulation-based training in improving crew skills.

Finally, regulation and compliance significantly affect Safety Performance. Hale et al. ^5^ emphasized the importance of a systemic approach to safety management, including strict regulations and periodic inspections. Knudsen ^40^ mentioned that the implementation of international regulations such as the SOLAS and ISM Code can improve safety, although low compliance rates in some countries are a challenge. ^38^

### 4) Key challenges faced by shipping companies in implementing Safety Performance

The main challenges in effectively implementing Safety Performance in the shipping industry include several aspects. First, lack of resources and funds is a major obstacle, especially for small shipping companies. Bloor and Sampson ^41^ noted that budget constraints often hinder the adoption of safety technology and crew training. Bhattacharya ^42^ added that investment in safety is often seen as a cost burden.

Second, regulatory inconsistencies and weak law enforcement are challenges. Knudsen ^40^ points out that compliance with safety regulations varies across countries, whereas Anderson ^43^ highlights the lack of coordination between international and national regulators as an obstacle to the effectiveness of safety policies.

Third, human factors and low safety awareness were obstacles. Hetherington et al. ^38^ found that many crew members were less aware of safety risks, whereas Bhattacharya and Tang ^44^ noted that crew members often prioritized operational efficiency over adherence to safety procedures.

Fourth, reliance on old technology and a lack of digitalization are additional obstacles. Rothblum et al. ^37^ noted that many ships still use outdated navigation systems, whereas Perera and Mo ^36^ highlighted resistance to new technologies as a barrier to implementation.

### 5) Adoption solutions to improve Safety Performance in Indonesian shipping companies

Solutions that can be adopted to improve the Safety Performance in Indonesian shipping companies include several strategic steps. Strengthening the safety culture is a priority, as recommended by Lu and Tsai ^3^ through the implementation of Safety Culture Indicators and Reason ^34^ with Just Culture to encourage open incident reporting.

Adoption of modern technology is also important. Ekstrand and Tapaninen ^4^ recommended the use of AIS, while Huang et al. ^6^ highlighted the role of big data analytics in predicting operational risks. Additionally, continuous training and simulations must be strengthened. Hetherington et al. ^38^ emphasized virtual reality-based simulations, whereas Schager ^39^ recommended scenario-based training to improve crew readiness. Collaboration and periodic inspections should be improved. Hale et al. ^5^ emphasized the need for regular safety audits to ensure compliance with safety standards.

### 6) The Role of Safety Performance in Creating Competitive Advantage for Shipping Companies

Safety Performance significantly contributes to the creation of competitive advantage in the shipping industry. First, the implementation of good Safety Performance improves the company's reputation. Lu and Tsai ^3^ show that shipping companies with a strong safety culture tend to have a better image in the eyes of customers and business partners. This directly impacts customer loyalty and the ability to attract more business contracts.

Second, companies with a good Safety Performance can reduce their operational costs. Rothblum et al. ^37^ found that the use of automated navigation systems reduces human error and the risk of accidents, which ultimately lowers incident-related costs such as ship repair costs and compensation. In addition, Hale et al. ^5^ stated that periodic safety inspections can minimize losses owing to equipment damage or unexpected operational stoppages.

Third, Safety Performance plays a role in increasing productivity and operational efficiency. Hetherington, Flin, and Mearns ^38^ emphasized that good safety training improves crew skills and readiness so that ship operations can run more smoothly. Big data-based predictive technology, as suggested by Perera and Mo, ^36^ helps companies optimize resource use through the real-time monitoring of ship conditions.

Fourth, from a sustainability perspective, good Safety Performance supports compliance with international regulations such as SOLAS and the ISM Code, which allow companies to operate in global markets. ^40^ Companies that comply with these regulations are also trusted by stakeholders, including customers, investors, and regulators.

Thus, Safety Performance is not only a tool to improve safety but also a strategic factor that supports the competitive advantage of shipping companies. Safety-first companies have an edge in terms of efficiency, reputation, and sustainability, all of which are essential for competing in the global marketplace.

Based on an analysis of the available literature, this study provides in-depth insights into the factors that influence Safety Performance in the shipping industry, including safety culture, technology, and human factors. A review of the Systematic Literature shows that Safety Performance has become a relevant topic in different regions with different focuses, such as research in the United States that focuses on health and transportation, as well as in China that highlights operational efficiency. In addition, the development of research discourse shows diverse theoretical approaches, including organizational, behavioral, and risk management theories. The main challenges in the implementation of Safety Performance are lack of resources, regulatory inconsistencies, and reliance on legacy technologies. Actionable solutions include strengthening safety culture, adoption of modern technology, and simulation-based training in Indonesian shipping companies.

However, the evidence included in this review has limitations, such as variations in the geographic context and methodology of the study. The review process is also limited by differences in measurement approaches and biases in reporting results. The implications of the results of this study for practice include the importance of improved crew training, the use of advanced technology, and stricter policies. Future research needs to focus on human factors and the application of new technologies in improving safety in the shipping sector, especially in developing countries such as Indonesia.

## 6. Conclusion

The results of this study show that Safety Performance has become an increasingly important and relevant topic in various sectors and global contexts. The literature trend analysis shows a significant increase in the number of publications, especially after 2000, with research predominating in developed countries, such as the United States and China. Research in this field covers a wide range of disciplines such as engineering, management, health, and economics, and highlights the important contributions of Business, Management, and Accounting.

Previous research has identified four main factors that affect Safety Performance in the shipping industry: safety culture, navigation technology and systems, human factors, and regulations and compliance. A strong safety culture, support of modern technologies such as AIS and big data analytics, ongoing training, and compliance with international regulations all contribute to reducing accident risks and improving operational safety.

However, the implementation of Safety Performance measures is challenging. Lack of resources and funding, regulatory inconformities, low safety awareness, and reliance on legacy technologies are the main obstacles. To address this, the proposed solutions include strengthening safety culture, adopting modern technology, simulation-based training, and periodic inspections to ensure compliance with safety standards.

Furthermore, Safety Performance not only improves safety but also creates a competitive advantage for shipping companies. With effective implementation, companies can improve their reputation, operational efficiency, and compliance with global sustainability standards, ultimately strengthening their positions in the international market.

## Ethics approval statement

This study is a systematic literature review that utilizes only previously published and publicly available data. As it does not involve human participants, animals, or primary data collection, ethical approval was not required.

## Data Availability

No data associated with this article. Repository name: **Data SLR Safety Performance on Competitive Advantage**. https://doi.org/10.6084/m9.figshare.28794725 ^45^ This project contains the following extended data:
• [Data SLR] (Collection of article data used for literature review) • [PRISMA flowchart] (A diagram illustrating the systematic process of selecting, screening, and including relevant articles for the literature review, based on predefined inclusion and exclusion criteria, following the PRISMA methodology) Figshare: PRISMA checklist for “The Influence of Safety Performance on Competitive Advantage: A Systematic Literature Review Study”. https://doi.org/10.6084/m9.figshare.28794725 ^45^ Data are available under the terms of the Creative Commons Attribution 4.0 International license (CC-BY 4.0).

## References

[1] Allianz Global Corporate & Specialty: *Global Claims Review 2022.* Allianz;2022.

[2] Grech MR Horberry T Koester T : *Human Factors in the Maritime Domain.* CRC Press;2008.

[3] Lu C-S Tsai C-L : The impact of safety culture on shipping safety performance. *Accid. Anal. Prev.* 2008;40(4):1258–1266.

[4] Ekstrand S Tapaninen U : The role of Automatic Identification System (AIS) in maritime safety and efficiency. *J. Navig.* 2020;73(2):1–15.

[5] Hale AR Borys D Adams M : Safety culture and safety management: Theoretical foundations and practical challenges. *Saf. Sci.* 2016;86:237–251.

[6] Huang C Chen Y Li X : The application of big data and predictive analytics in maritime safety. *International Journal of Shipping and Logistics.* 2021;13(3):245–258.

[7] Kitchenham B Brereton OP Budgen D : Systematic literature reviews in software engineering–a systematic literature review. *Inf. Softw. Technol.* 2009;51(1):7–15. 10.1016/j.infsof.2008.09.009

[8] Borret SR Sheble L Moody J : Bibliometric review of ecological network analysis: 2010-2016. 2018.

[9] Wahono RS : A systematic literature review of software defect prediction. *Journal of Software Engineering.* 2015;1(1):1–16.

[10] Ernawati N : *Buku Ajar Mata Kuliah Metodologi Riset Penelitian Data Sekunder.* Soepraoen, Malang: Poltekkes RS dr;2020.

[11] Delgado-Rodríguez M Sillero-Arenas MJMI : Systematic review and meta-analysis. *Medicina Intensiva (English Edition).* 2018;42(7):444–453. 10.1016/j.medine.2017.10.012 29169792

[12] Khairunnisa A Juandi D Gozali SM : Systematic literature review: Kemampuan pemahaman matematis siswa dalam menyelesaikan masalah matematika. *Jurnal Cendekia.* 2022;6(2):1846–1856. 10.31004/cendekia.v6i2.1405

[13] Sari EP : *Sistematik Literatur Review Jenis Pohon Pelindung Jalan Dari Tahun 2007-2023 Sebagai Sumber Belajar Biologi.*(Undergraduate thesis, Universitas Muhammadiyah Malang).2024.

[14] Gnoni MG Saleh JH : Near-miss management systems and observability-in-depth: Handling safety incidents and accident precursors in light of safety principles. *Saf. Sci.* 2017;91:154–167. 10.1016/j.ssci.2016.08.012

[15] Brand LM : Exploring a model of psychological fitness for work: are individual difference variables relevant in a model of safety performance? 2010. Doctoral dissertation.

[16] Razali NA Redzuan NIN Kamaruddin AN : A study on safety management practices and safety performance. *Eur. Proc. Soc. Behav. Sci.* 2018;44. 10.15405/epsbs.2018.07.02.27

[17] Griffin MA Neal A : Perceptions of Safety at Work: A Framework for Linking Safety Climate to Safety Performance, Knowledge, and Motivation. *J. Occup. Health Psychol.* 2000;5(3):347–358. 10.1037/1076-8998.5.3.347 10912498

[18] Hon CKH Chan APC Yam MCH : Relationships Between Safety Climate and Safety Performance of Building Repair, Maintenance, Minor Alteration, and Addition (RMAA) Works. *Saf. Sci.* 2014;65:10–19. 10.1016/j.ssci.2013.12.012

[19] Wu TC Chang SH Shu CM : Safety leadership and safety performance in petrochemical industries: The mediating role of safety climate. *J. Loss Prev. Process Ind.* 2011;24(6):716–721. 10.1016/j.jlp.2011.04.007

[20] Muhieddine MH Viswanath SK Armstrong A : Model-based solvent selection for the synthesis and crystallisation of pharmaceutical compounds. *Chem. Eng. Sci.* 2022;264:118125. 10.1016/j.ces.2022.118125

[21] Ashraf MT Dey K Pyrialakou D : Investigation of pedestrian and bicyclist safety in public transportation systems. *J. Transp. Health.* 2022;27:101529. 10.1016/j.jth.2022.101529

[22] Zhang Y Zhou YD Wu H : Influence of water level on RC caisson subjected to underwater explosions. *Ocean Eng.* 2022;266:113162. 10.1016/j.oceaneng.2022.113162

[23] Zhong X Tian P Chen C : Preparation and Interface Stability of Alginate-based Gel Polymer Electrolyte for Rechargeable Aqueous Zinc Ion Batteries. *J. Electroanal. Chem.* 2022;116968.

[24] Erten B Oral B Yakut MZ : The role of virtual and augmented reality in occupational health and safety training of employees in PV power systems and evaluation with a sustainability perspective. *J. Clean. Prod.* 2022;379:134499. 10.1016/j.jclepro.2022.134499

[25] Rojas OE Khan MA : A review on electrical and mechanical performance parameters in lithium-ion battery packs. *J. Clean. Prod.* 2022;378:134381. 10.1016/j.jclepro.2022.134381

[26] Schürmann B Klischat M Kochdumper N : Formal safety net control using backward reachability analysis. *IEEE Trans. Autom. Control.* 2021;67(11):5698–5713.

[27] Ricker M Feiri T Nille-Hauf K : Contribution to efficient structural safety assessments: A comparative analysis of computational schemes. *Probabilistic Eng. Mech.* 2022;69:103285. 10.1016/j.probengmech.2022.103285

[28] Russell S Bennett J Wellman JA : Efficacy and safety of voretigene neparvovec (AAV2-hRPE65v2) in patients with RPE65-mediated inherited retinal dystrophy: a randomised, controlled, open-label, phase 3 trial. *Lancet.* 2017; vol.390(10097): pp.849–860. 10.1016/S0140-6736(17)31868-8 28712537 PMC5726391

[29] Gore ME Szczylik C Porta C : Safety and efficacy of sunitinib for metastatic renal-cell carcinoma: an expanded-access trial. *Lancet Oncol.* 2009;10(8):757–763. 10.1016/S1470-2045(09)70162-7 19615940

[30] Niskanen T : Safety climate in organizational settings: Changes and challenges. *Saf. Sci.* 1994;17(2):237–255. 10.1016/0925-7535(94)90026-4

[31] Vinodkumar MN Bhasi MJSS : Safety climate factors and its relationship with accidents and personal attributes in the chemical industry. *Saf. Sci.* 2009;47(5):659–667. 10.1016/j.ssci.2008.09.004

[32] Neal A Griffin MA Hart PM : The role of knowledge, motivation, and safety climate in safety performance. *J. Appl. Psychol.* 2000;85(3):281–293.

[33] Xia N Zou PX Liu X : A hybrid BN-HFACS model for predicting safety performance in construction projects. *Saf. Sci.* 2018;101:332–343. 10.1016/j.ssci.2017.09.025

[34] Reason J : *Managing the Risks of Organizational Accidents.* Routledge;1997.

[35] Ek Å Akselsson R Arvidsson M : Safety culture in Swedish air traffic control. *Saf. Sci.* 2000;34(1-3):67–87.

[36] Perera LP Mo B : Marine ship intelligence for autonomous navigation: Big data and artificial intelligence approach. *Ocean Eng.* 2016;126:57–76.

[37] Rothblum AM Wheal D Withington S : Human error and marine safety, U.S. Coast Guard Research Report. 2002.

[38] Hetherington C Flin R Mearns K : Safety in shipping: The human element. *J. Saf. Res.* 2006;37(4):401–411. 10.1016/j.jsr.2006.04.007 17046789

[39] Schager B : The importance of training and simulation in maritime safety. *Maritime Safety Training Journal.* 2008;8(3):145–157.

[40] Knudsen F : Paperwork at the service of safety? Workers’ reluctance against written procedures exemplified by the concept of ‘seamanship’. *Saf. Sci.* 2009;47(2):295–303. 10.1016/j.ssci.2008.04.004

[41] Bloor M Sampson H : Regulatory enforcement of occupational health and safety at sea: The role of Port State Control. *Sociol. Health Illn.* 2009;31(5):709–725.

[42] Bhattacharya S : The effectiveness of the ISM Code: A qualitative enquiry. *Mar. Policy.* 2012;36(2):528–535. 10.1016/j.marpol.2011.09.004

[43] Anderson P : International coordination and maritime safety: Challenges in achieving global standards. *Marit. Policy Manag.* 2003;30(2):165–176.

[44] Bhattacharya S Tang L : Fatigue, sleep and shift work in the maritime industry. *Health and Safety Executive Research Report.* 2013.

[45] Raharjo BA Utami HN Afrianty TW : Data SLR Safety Performance on Competitive Advantage. *figshare.* 2025. 10.6084/m9.figshare.28794725 PMC12280866 40698025

