# Supplementary material for: The Influence of Safety Performance on Competitive Advantage in the Shipping Industry: A Systematic Literature Review Study
Source: F1000Res. 2026 Mar 5;14:490. Originally published 2025 May 12. [Version 4] doi: 10.12688/f1000research.163658.4 (PMC12280866; doi:10.12688/f1000research.163658.4)
Supplement: Peer review history

# Reviewer report: Reviewer response for version 4

**DOI:** 10.5256/f1000research.197202.r464956

**Published:** 2026-04-16

**Authors:** Benny Hutahayan

**Affiliations:** 1 University of Brawijaya, Brawijaya, Indonesia

You have revised your manuscript according to the reviewer's suggestions.

 Good luck

Are the rationale for, and objectives of, the Systematic Review clearly stated?

Yes

Is the statistical analysis and its interpretation appropriate?

Yes

If this is a Living Systematic Review, is the ‘living’ method appropriate and is the search schedule clearly defined and justified? (‘Living Systematic Review’ or a variation of this term should be included in the title.)

Yes

Are sufficient details of the methods and analysis provided to allow replication by others?

Partly

Are the conclusions drawn adequately supported by the results presented in the review?

Yes

Reviewer Expertise:

Business Administration, Human Resources Management, Law, Finance

I confirm that I have read this submission and believe that I have an appropriate level of expertise to confirm that it is of an acceptable scientific standard.

# Reviewer report: Reviewer response for version 3

**DOI:** 10.5256/f1000research.187229.r409723

**Published:** 2026-02-26

**Authors:** Benny Hutahayan

**Affiliations:** 1 University of Brawijaya, Brawijaya, Indonesia

The manuscript has already been revised well in Version 2, and based on that revision, my decision is to approve the article.

Are the rationale for, and objectives of, the Systematic Review clearly stated?

Yes

Is the statistical analysis and its interpretation appropriate?

Yes

If this is a Living Systematic Review, is the ‘living’ method appropriate and is the search schedule clearly defined and justified? (‘Living Systematic Review’ or a variation of this term should be included in the title.)

Yes

Are sufficient details of the methods and analysis provided to allow replication by others?

Partly

Are the conclusions drawn adequately supported by the results presented in the review?

Yes

Reviewer Expertise:

Business Administration, Human Resources Management, Law, Finance

I confirm that I have read this submission and believe that I have an appropriate level of expertise to confirm that it is of an acceptable scientific standard.

# Response

**Published:** 2026-02-26

**Authors:** Bharto Ari Raharjo

**Affiliations:** Brawijaya University, Malang, East Java, Indonesia

Thank you for your comments. However, we would like to respectfully clarify that this manuscript is a Systematic Literature Review (SLR), not an empirical cross-sectional study. Therefore, it does not formulate hypotheses (H1, H2, H3, etc.) and does not require tests such as common method bias (CMB). Our study is guided by research questions, not hypothesis testing.

# Reviewer report: Reviewer response for version 2

**DOI:** 10.5256/f1000research.183274.r390333

**Published:** 2025-08-23

**Authors:** Elriza Esterhuyzen

**Affiliations:** 1 University of South Africa, Pretoria, Gauteng, South Africa

The article is of good quality and provides valuable information applicable to the shipping industry. It is suggested that the shipping industry be mentioned in the title, as the current title is slightly misleading - one would expect to find general information on safety performance and competitive advantage, not only in the shipping industry in Indonesia. The generalizability of findings is thus limited.

 The information used (Scopus data up until 2022) seems outdated. It is not clear why the SLR ended in 2022 - why not include it until 2025? There are also some discrepancies in text as mention is made of a 2023 article (for example) which falls outside the scope previously mentioned.

 It is also concerning that mention is made that no ethical approval was required as this is still a requirement, even for SLRs.

Are the rationale for, and objectives of, the Systematic Review clearly stated?

Yes

Is the statistical analysis and its interpretation appropriate?

Partly

If this is a Living Systematic Review, is the ‘living’ method appropriate and is the search schedule clearly defined and justified? (‘Living Systematic Review’ or a variation of this term should be included in the title.)

Not applicable

Are sufficient details of the methods and analysis provided to allow replication by others?

Yes

Are the conclusions drawn adequately supported by the results presented in the review?

Yes

Reviewer Expertise:

Occupational Safety and Health, Business Management, Entrepreneurship, Risk Management, Sustainability

I confirm that I have read this submission and believe that I have an appropriate level of expertise to confirm that it is of an acceptable scientific standard, however I have significant reservations, as outlined above.

# Response

**Published:** 2025-08-25

**Authors:** Bharto Ari Raharjo

**Affiliations:** Brawijaya University, Malang, East Java, Indonesia

Dear Reviewer,

 Thank you very much for your constructive comments and suggestions, which have helped us improve the quality and clarity of our manuscript. We want to address your concerns as follows:

 We have revised the title to explicitly reflect the shipping industry context, to avoid any misleading interpretation.

 We sincerely apologize for the inconsistency regarding the data coverage period. We would like to clarify that the Scopus data used in this study were collected up to 2023. The reason the literature search ended in 2023 is that the systematic review was conducted in 2024.

 Therefore, the dataset reflects the latest available publications at that time. We have corrected this point throughout the manuscript to ensure consistency and accuracy.

 However, our study is a systematic literature review that relies exclusively on secondary data from previously published journal articles. Therefore, no direct involvement of human participants or animals was undertaken in this research. In accordance with international ethical standards, ethical approval is mandatory for studies involving living subjects. To avoid any misunderstanding, we have revised the manuscript accordingly. We have clarified the ethical statement to explicitly mention this.

# Reviewer report: Reviewer response for version 2

**DOI:** 10.5256/f1000research.183274.r392589

**Published:** 2025-07-21

**Authors:** Alina Trifu

**Affiliations:** 1 Occupational Risks Assessment and Management of Health and Safety at Work Laboratory, National Research and Development Institute for Occupational Safety INCDPM Bucharest, Bucharest, Romania

Thank you very much for the opportunity to read and review your article titled: „The Influence of Safety Performance on Competitive Advantage: A Systematic Literature Review Study”

 This manuscript addresses a significant topic: safety performance in the shipping safety context.

 This study analyzes research trends and developments related to Safety Performance and its implementation solutions through the Systematic Literature Review based on Scopus data from 1919 to 2022.

 The rationale and the objectives of the Systematic Review are clearly stated. All the specialized terms used are very well described. There are sufficient details of the methods used for systematic review to allow replication. The conclusions are drawn adequately and are supported by the results presented in the review. The limitations of the work are well acknowledged.

 I recommend indexing the article.

Are the rationale for, and objectives of, the Systematic Review clearly stated?

Yes

Is the statistical analysis and its interpretation appropriate?

Yes

If this is a Living Systematic Review, is the ‘living’ method appropriate and is the search schedule clearly defined and justified? (‘Living Systematic Review’ or a variation of this term should be included in the title.)

Not applicable

Are sufficient details of the methods and analysis provided to allow replication by others?

Yes

Are the conclusions drawn adequately supported by the results presented in the review?

Yes

Reviewer Expertise:

Occupational Safety, Occupational Risks Assessment, Health and Safety

I confirm that I have read this submission and believe that I have an appropriate level of expertise to confirm that it is of an acceptable scientific standard.

# Reviewer report: Reviewer response for version 2

**DOI:** 10.5256/f1000research.183274.r389972

**Published:** 2025-06-12

**Authors:** Benny Hutahayan

**Affiliations:** 1 University of Brawijaya, Brawijaya, Indonesia

Thank you for the revisions that have been made. After reviewing them, I appreciate your efforts in thoroughly addressing all the feedback and revising the manuscript in accordance with the suggestions provided. The changes have significantly improved the quality of the manuscript, both in terms of substance and clarity of writing. I also noticed that Figures 2, 3, 4, and 5 have been revised.

Are the rationale for, and objectives of, the Systematic Review clearly stated?

Yes

Is the statistical analysis and its interpretation appropriate?

Yes

If this is a Living Systematic Review, is the ‘living’ method appropriate and is the search schedule clearly defined and justified? (‘Living Systematic Review’ or a variation of this term should be included in the title.)

Yes

Are sufficient details of the methods and analysis provided to allow replication by others?

Partly

Are the conclusions drawn adequately supported by the results presented in the review?

Yes

Reviewer Expertise:

Business Administration, Human Resources Management, Law, Finance

I confirm that I have read this submission and believe that I have an appropriate level of expertise to confirm that it is of an acceptable scientific standard.

# Response

**Published:** 2025-06-16

**Authors:** Bharto Ari Raharjo

**Affiliations:** Brawijaya University, Malang, East Java, Indonesia

Dear Reviewers,

 I would like to express my sincere thanks for your valuable feedback on my manuscript. I greatly appreciate the time and effort you invested in reviewing my work. Your suggestions have been immensely helpful in improving the quality of the manuscript.

 Thank you once again for your support.

 Best regards,

# Reviewer report: Reviewer response for version 1

**DOI:** 10.5256/f1000research.180046.r384675

**Published:** 2025-05-27

**Authors:** Benny Hutahayan

**Affiliations:** 1 University of Brawijaya, Brawijaya, Indonesia

I have read the entire manuscript; overall, it is well written and coherent. The manuscript is also easy to understand for readers. However, I have several comments, notes, suggestions, and potential revisions that may help improve the quality of the manuscript:

1. The introduction is written systematically and flows well. I believe this section is already appropriate.
2. In the literature review section, please consider adding references related to "Systematic Literature Review" and "Safety Performance." Since this is a literature review section, it currently includes only a limited number of sources. The manuscript would benefit greatly from incorporating various perspectives in this part.
3. The syntax used for data collection should be italicized to avoid potential misunderstandings by readers.
4. The inclusion criteria are explained in the research method section, but the exclusion criteria are only briefly mentioned. I recommend adding one sentence that explicitly states the exclusion criteria.
5. In the discussion section (Figures 2, 3, 4, 5), it is mentioned "on the right side of the figure" and "on the left side of the figure," but I can only see one graph/diagram in each case. Please review and revise this, as some figures may be missing. Aside from this issue, the discussion is quite appropriate.
6. The conclusion is well-written; however, before finalizing my assessment, I would like to see how the missing figures (as mentioned in point 5) are addressed.

Are the rationale for, and objectives of, the Systematic Review clearly stated?

Yes

Is the statistical analysis and its interpretation appropriate?

Yes

If this is a Living Systematic Review, is the ‘living’ method appropriate and is the search schedule clearly defined and justified? (‘Living Systematic Review’ or a variation of this term should be included in the title.)

Yes

Are sufficient details of the methods and analysis provided to allow replication by others?

Partly

Are the conclusions drawn adequately supported by the results presented in the review?

Yes

Reviewer Expertise:

Business Administration, Human Resources Management, Law, Finance

I confirm that I have read this submission and believe that I have an appropriate level of expertise to confirm that it is of an acceptable scientific standard, however I have significant reservations, as outlined above.
